# Proteomics Analysis of Pregnancy in Ewes under Heat Stress Conditions and Melatonin Administration

**DOI:** 10.3390/ani14030400

**Published:** 2024-01-26

**Authors:** Efterpi Bouroutzika, Stavros Proikakis, Ekaterini K. Theodosiadou, Konstantinos Vougas, Angeliki I. Katsafadou, George T. Tsangaris, Irene Valasi

**Affiliations:** 1 Faculty of Veterinary Science, University of Thessaly, 43131 Karditsa, Greece; bouroutz@uth.gr (E.B.); etheodosiadou@uth.gr (E.K.T.); 2 Laboratory of Food Quality Control and Hygiene, Department of Food Science and Human Nutrition, Agricultural University of Athens, 11855 Athens, Greece; stavrospro95@yahoo.gr; 3 Proteomics Research Unit, Biomedical Research Foundation of the Academy of Athens, 11527 Athens, Greece; kvougas@bioacademy.gr (K.V.), gthtsangaris@bioacademy.gr (G.T.T.); 4 Faculty of Public and One Health, University of Thessaly, 43100 Karditsa, Greece; agkatsaf@uth.gr

**Keywords:** melatonin, heat stress, pregnancy, parturition, proteomics, ewes

## Abstract

**Simple Summary:**

Heat stress is known to cause disruptions to a variety of physiological processes in sheep, including reproduction and ultimately pregnancy, mostly by promoting reactive oxygen species generation and oxidative stress. It has been proposed that the exogenous administration of melatonin, because of its antioxidant and immunomodulatory properties, could help alleviate adverse effects of heat stress. However, the way that melatonin modulates these activities is unclear, especially during pregnancy under heat stress conditions, as well as during lambing. For these reasons, proteomics analysis was used to elucidate the proteins that are regulated by the presence of melatonin. The findings derived from proteomics analysis indicated that melatonin regulates proteins that are involved in cell cycle division, boosted immune response and protective mechanisms affecting both the maternal organism and the embryo(s).

**Abstract:**

Melatonin is an indoleamine with broad spectrum properties that acts as a regulator of antioxidant and immune response in organisms. In our previous studies, melatonin improved redox status and inflammatory response in pregnant ewes under heat stress conditions. In the present study, using proteomics, the proteins regulated by melatonin during different stages of pregnancy and lambing were assessed. Twenty-two ewes equally divided into two groups, the melatonin (M) (n = 11) and control (C) group (n = 11), participated in the study and were exposed to heat stress during the first months of pregnancy. In the M group, melatonin implants were administered throughout pregnancy, every 40 days, until parturition (a total of four implants per ewe). Blood samples were collected at the beginning of the study simultaneously with the administration of the first melatonin implant (blood samples M1, C1), mating (M2, C2), second implant (M3, C3), fourth implant (M4, C4) and parturition (M5, C5), and MALDI-TOF analysis was performed. The results revealed the existence of 42 extra proteins in samples M2, M3 and M4 and 53 in M5 (sample at parturition) that are linked to melatonin. The biological processes of these proteins refer to boosted immune response, the alleviation of oxidative and endoplasmic reticulum stress, energy metabolism, the protection of the maternal organism and embryo development. This proteomics analysis indicates that melatonin regulates protective mechanisms and controls cell proliferation under exogenous or endogenous stressful stimuli during pregnancy and parturition.

## 1. Introduction

A series of events that include oocyte maturation, fertilization, blastocyst implantation in the uterus and embryo development are necessary for a successful pregnancy. However, a stressor stimulus, including environmental changes with extreme weather and temperature conditions, can lead to pregnancy failure by disrupting one or more of these processes. The majority of mechanisms leading to embryonic loss include excessive reactive oxygen species (ROS) generation which promotes oxidative stress, which cannot be reversed by the maternal antioxidant system [1]. Sheep reared in the Mediterranean region are exposed to heat stress during summer months, a period when most of them establish pregnancy. High ambient temperatures are responsible for extended oxidative stress on animals, causing disruptions in pregnancy [2]. Heat stress has been found to exert attenuating effects on gene expression related to follicular growth, steroid secretion, the elongation of the estrous cycle and a reduction in estrus duration and intensity [3]. To minimize the complications that heat stress causes to the organism, heat shock proteins (HSPs) are activated as protective agents for the cell. HSPs exhibit chaperone activity, which is essential for controlling the folding, unfolding and refolding of stress-denatured proteins [4]. However, their activation is not enough for reversing the harmful effects of heat stress.

For this reason, new strategies for oxidative stress alleviation have been suggested and tested. One of them proposes melatonin administration during the summer period, based on the antioxidant and anti-inflammatory/immunomodulatory properties that this neurohormone exerts [5,6] apart from the regulation of the circadian cycle and sleep. Recently, we found that melatonin administration throughout pregnancy enhanced redox status in heat-stressed pregnant ewes, mean number and bodyweight of lambs born per ewe, as well as milk production [7], while furthermore exerting immunomodulatory and antioxidant action on their newborn offspring [8]. Melatonin is an indolamine with an amphiphilic nature and acts as a free radical scavenger or upregulates antioxidant gene expression such as the glutathione peroxidase (GPx) gene. Melatonin’s activity as a scavenger or regulator molecule depends on its concentration and the number of its receptors in each cell compartment [9]. The two major membrane receptors are ΜΤ1 and ΜΤ2, which belong to the G-coupled receptor protein family with seven transmembrane domains and are located in all organs [10]. The effect of melatonin is based on the number of its receptors, nuclear binding sites and their interactions with cytosolic molecules [11]. The nuclear binding sites are involved in melatonin’s inhibition of inflammation [12] and in the stimulation of antioxidant enzymes [13]. The effects of melatonin that are attributed to its interaction with calmodulin include the regulation of the cytoskeleton, as reported by Benitez-King and Anton-Tay [14], and the modulation of neuronal nitric oxide synthase, as indicated by Pozo et al. [15]. Moreover, the influence of melatonin on the cytoskeleton involves membrane receptors [14], while the intracellular actions of melatonin, both in the cytosol and the nucleus, are facilitated by its ability to easily traverse cell membranes, providing melatonin access to various subcellular organelles [16]. All the latter mechanisms highlight the capacity of melatonin to ameliorate oxidative stress. Furthermore, studies have shown that melatonin plays a bidirectional role in the immune system, acting as a buffer to regulate innate and other specific immune responses [17]. Melatonin enhances immune functions through two main mechanisms: increasing antigen presentation to immune cells, thereby boosting antibody production, and regulating cytokine production to control cellular responses [18]. Specifically, melatonin administration enhances cytokine production by increasing the proliferation of B cells and the release of cytokines associated with the T helper cells type 1 (Th1) immune response, such as interleukin (IL)-2 and interferon-gamma (IFN-γ). Additionally, melatonin administration suppresses the production of Th2 cytokines like IL-10 [19].

Protein identification in biological samples is performed by means of proteomics, creating the missing bridge among genome, transcriptome and biological function [20]. The most commonly used proteomic methods include 2D gel electrophoresis, followed by MALDI-TOF-MS (Matrix-Assisted Laser Desorption/Ionization Time-of-Flight Mass Spectrometry) and HPLC-MS/MS (High-Performance Liquid Chromatography coupled with Tandem Mass Spectrometry). All the obtained proteins are compared to reference databases, in order to ensure the accuracy of results [21,22,23,24,25]. Based on all the information that proteomics provides for the structure, function, and regulation of proteins, it is the ideal tool to shed light on all interactions between melatonin and proteins involved in the cell’s response to oxidative stress during gestation. Currently, there is a significant lack of knowledge in the field of proteomics analysis during pregnancy and after melatonin administration in both animals and humans. Thus, the present study was designed to elucidate and confirm at the molecular level our initial hypothesis, which has already been studied in pregnant ewes [7], by means of proteomics, that melatonin may act as an antioxidant and immunomodulatory regimen throughout the reproductive process, from mating to pregnancy and finally to parturition. The aim of this study was to screen the proteome of ewes exposed to heat stress during the first months of pregnancy due to high ambient temperatures in summer and the influence that melatonin administration exerts on proteins at mating and from early pregnancy until parturition.

## 2. Materials and Methods

### 2.1. Experimental Overview

In total, twenty-two pregnant ewes of the Karagkouniko breed, aged 2–4 years, were selected within a flock of ewes after estrous synchronization and pregnancy diagnosis to participate in the proteomics analysis. The animals were randomly allocated to two equal groups: the M group received melatonin implants (n = 11) and the C group served as the control animals (n = 11).

The study began within the breeding season of the breed, in July in central Greece (Thessaly; 52°43′52″ N), and lasted until the lambing period, which took place in early January. A protocol for estrous synchronization was applied to all ewes followed by natural mating, while melatonin was administered only in ewes of the M group, as previously described, and evaluated by Bouroutzika et al. [7]. Briefly, in the ewes of the M group, a total of four melatonin implants (dose rate: 1 implant per ewe; Regulin, Ceva, Lisbourne, France) were inserted, one implant every 40 days (D0, D40, D80, D120), starting on the day of the insertion of progestogen sponges for estrous synchronization (D0). Pregnancy diagnosis and pregnancy confirmation were performed by means of ultrasonographic examination (U/S) at 60 and 84 days, respectively, after the beginning of the experimental period. In all pregnant ewes that participated in the study, pregnancy and parturition proceeded normally. The same health management practices (vaccinations, anthelminthic treatment and diet) were consistently applied to all ewes during the experimental period.

The ewes were housed throughout the study. Daily measurements of temperature and humidity inside and outside the animal facilities were taken starting on day 0 (D0) using a portable temperature data logger (HD 32.2, Delta OHM, Caselle di Selvazzano, Italy). To monitor the environmental conditions within the animal facility, the logger was placed on a table for six hours daily between 10:00 and 16:00. To calculate the Temperature–Humidity Index (THI), a useful metric for assessing heat stress severity, the following formula was applied, as it was stated by Marai et al. [26] and Habeeb et al. [27]:

THI = db °C − [(0.31 − 0.31 RH) × (db °C − 14.4)], where db °C is the dry-bulb temperature (°C) and RH is the relative humidity provided by the logger. To interpret the results, the average of the two THI values at 10:00 and 16:00 was calculated. The THI values were then classified based on the standard provided by Marai et al. [26]:

<22.20: no heat stress.

22.20–23.29: mild heat stress.

23.30–25.59: severe heat stress.

≥25.60: extreme severe heat stress.

Additionally, rectal temperature and breathing rate were measured twice daily in all animals in each group, at 10:00 and 16:00. The recordings of THI, rectal temperature and breathing rate terminated ten days after THI first entered the zone below 22.20.

**Figure 1:**
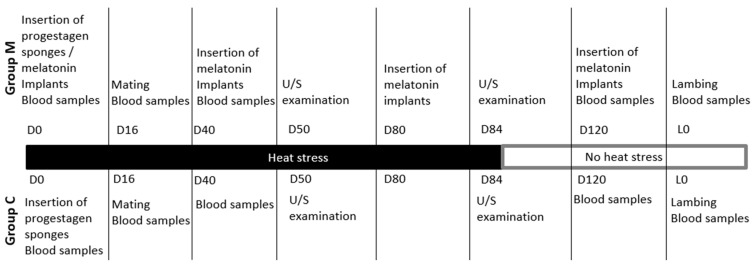
Timeline of the study.

A detailed timeline is presented in Figure 1.

### 2.2. Sampling and Preparation of Samples

Blood samples were collected from all ewes via jugular venipuncture (EDTA, BD Vacutainer^®^ blood collection tubes, Franklin Lakes, NJ, USA) at D0, D16, D40 and D120 and at lambing (L0); plasma samples were stored at −80 °C until they were assayed by means of proteomics. Samples collected at each of the 5 time points (i.e., D0, D16, D40, D120 and L0) and from all ewes in each group (i.e., groups M and C) were pooled to form the samples M1–M5 and C1–C5 for groups M and C, respectively. In total, 13 samples were analyzed in duplicates as follows: the samples M1 and C1 were pooled together (sample M1–C1), the samples M2, M3, M4 and M5 and C2, C3, C4 and C5 were assayed separately, and, additionally, the samples M3, C3, M5 and C5 were assayed after applying the ProteoMiner^TM^ enrichment kit (Bio-Rad, Hercules, CA, USA).

### 2.3. ProteoMiner^TM^ Enrichment Kit

The ProteoMiner^TM^ enrichment kit (Bio-Rad, Hercules, CA, USA) was employed following the manufacturer’s guidelines. To prepare the columns, the top and bottom caps were first removed, and the columns were then centrifuged for 30–60 s to eliminate any storage material, with the resulting flow-through being discarded. Following this, 200 μL of wash buffer was added to the column, and the column was rotated for 5 min. Subsequently, it was centrifuged again for 30–60 s, and once more, the flow-through was discarded. These same steps were repeated for a second wash with the wash buffer. Once the column was properly prepared, 200 μL of plasma sample with a protein concentration of ≥50 mg/mL was added to the column. It was then rotated at room temperature for 2 h and subsequently centrifuged for 30–60 s. The filtrate containing the high-abundance proteins was discarded. The centrifugation step was repeated, and another 200 μL of wash buffer was added to the column. After 5 min of rotation, the column was centrifuged again for 30–60 s, and the resulting filtrate was once more discarded. This final step with 200 μL of wash buffer was repeated three times.

### 2.4. Two-Dimensional Gel Electrophoresis

In brief, the protein concentration was measured via Bradford assay, and 750 μg of total plasma protein from non-depleted samples was loaded on 18 cm immobilized ReadyStripTM IPG gradient strips with a pH range of 3–10 NL (Bio-Rad, Hercules, CA, USA). Strips were rehydrated with rehydration buffer (8 M urea, 2% CHAPS and 0.4% DTE) overnight before samples were loaded. The next day, the strips were transferred to an IPGphor isoelectric focusing system with focusing starting at 250 V for 30 min, after which the voltage was linearly increased to 5000 V for 20 h and then rapidly increased to 5000 V for another 12 h. After first-dimension electrophoresis, each of the strips was treated with a solution consisting of 12.5 mL Tris-HCl 1.5 M pH 8.8, 20 mL of acrylamide solution 30% SDS, 17 mL of distilled water and 500 μL of SDS solution 20%. After adding 500 μL APS 10% and 50 μL TEMED (Bio-Rad, Hercules, CA, USA), the polymerization process began to create 12% SDS-polyacrylamide gels (180 × 200 × 1.5 mm^3^) for second-dimension electrophoresis. The vertical electrophoresis was carried out with a run of 40 mA/gel, using PROTEIN-II multicell apparatuses (Bio-Rad, Hercules, CA, USA). The gels were fixed in 50% methanol containing 5% phosphoric acid overnight. The fixative solution was washed off by agitation in distilled water for 45 min. The gels were stained with colloidal Coomassie blue (Novex, San Diego, CA, USA), scanned in a GS-800 Calibrated Densitometer (Bio-Rad, Hercules, CA, USA) using the PD-Quest v8.0 2-DE analysis software (Bio-Rad, Hercules, CA, USA) and stored on computer for further analysis. Gel reproducibility was assessed by running duplicate gels of each protein extract. The procedure is described by Tsangaris et al. and Scoppetta [28,29]. Gel images were analyzed according to Zografos et al. [30] and Katsafadou et al. [31]. All gels’ protein spots were analyzed and were detected, aligned and matched using the PD-Quest v8.0 image processing software, according to the manufacturer’s instructions. Manual inspection of the spots was used for the verification of matching accuracy.

### 2.5. Peptide Mass Fingerprint (PMF)

Gel spots of interest were manually annotated using Melanie 4.02 software and excised from 2-DE gels using Proteiner SPII (Bruker Daltonics, Bremen, Germany), destained with 30% acetonitrile in 50 mM ammonium bicarbonate and dried in a speed vacuum concentrator (MaxiDry Plus, Heto, Allered, Denmark), and a 16 h trypsin (Roche Diagnostics, Basel, Switzerland) digestion was performed. MS analyses were conducted on a MALDI-MS MS in a time-of-flight mass spectrometer (Ultraflex II, Bruker Daltonics, Bremen, Germany). The detailed procedure is described by Kolialexi et al. [32].

Peptide matching and protein searches were performed automatically with Mascot server 2 (Matrix Sciences, London, UK). Peptide masses were compared with the theoretical peptide masses of all available proteins from *Ovies aries/Ruminantia* in the UniProtKB database (unreviewed (TrEMBL) and reviewed (Swiss-Prot); version 2013_04). Stringent criteria were used for protein identification with a maximum allowed mass error of 25 ppm and a minimum of 4 matching peptides. Probability score with *p* < 0.05 was the threshold for affirmative protein identification. Monoisotopic masses were used, and one missed trypsin cleavage site was calculated for proteolytic products. The searching parameters included the potential alteration of the residue mass due to the possible existence of carbamidomethylation and oxygenation. Redundant proteins found in databases with different names and accession numbers were eliminated. If more than one protein was identified under one spot, the single protein member with the highest protein score was singled out from the multi-protein family. A Mascot score > 40 and an expression level of >1.5 or <0.5 were used. Mascot scores >40 indicated identity or extensive homology at the *p* < 0.05 level. The above bases were used to determine the biological processes of proteins.

## 3. Results

### 3.1. Clinical Signs and THI Results

**Figure 2:**
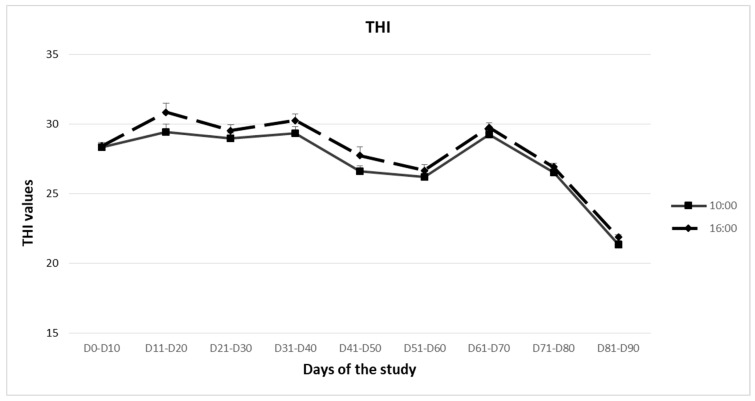
Mean Temperature–Humidity Index (THI) values calculated daily at 10:00 and 16:00 during the first 90 days of the study.

Mean THI was higher than 25.60 in both the 10:00 and 16:00 measurements throughout the first 80 days of the study, indicating “extreme severe heat stress” conditions (Figure 2). During the period from D81 to D90, which coincided with the middle of October, THI declined below 22.20, signaling the beginning of the non-heat stress period for the ewes.

Mean rectal temperature and breathing rate (Table 1) were elevated in both 10:00 and 16:00 measurements throughout the first 80 days of the study. During the period from D81 to D90, the clinical signs returned within the normal range for the breed and animal species.

### 3.2. Proteomics Analysis Results

The sample M1–C1 was the control sample of the study prior to melatonin treatment and pregnancy establishment. In samples M1–C1 and C5, whole mapping was performed, as well as in samples C3 and C5 after applying the ProteoMiner^TM^ enrichment kit. The results revealed the presence of 186 proteins in the sample M1–C1, 63 proteins in the C3 sample after applying the ProteoMiner^TM^ enrichment kit, as well as 263 and 113 before and after applying the ProteoMiner^TM^ enrichment kit in the C5 sample at parturition, respectively. The catalogue of proteins derived from whole mapping is available in the Supplementary Materials.

**Figure 3:**
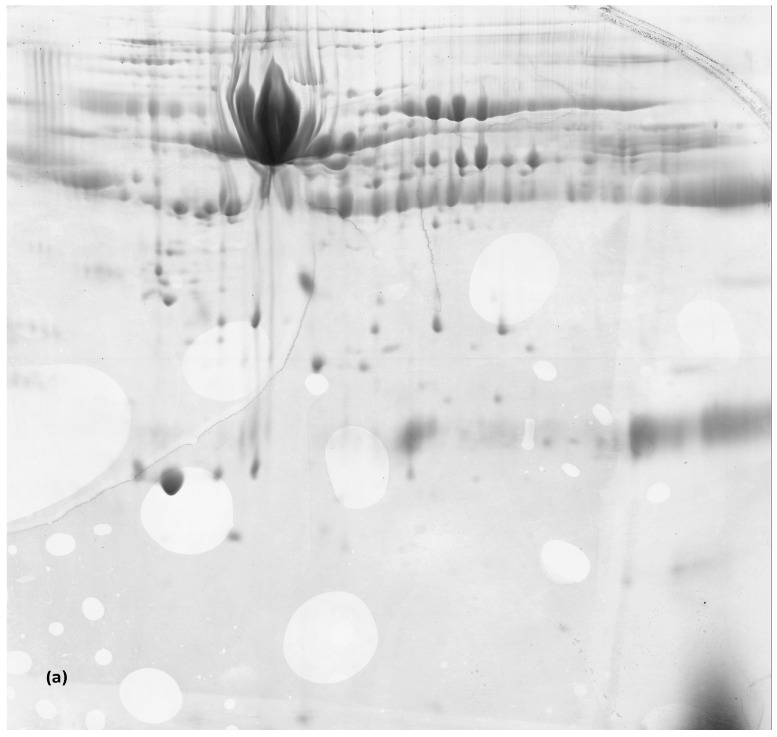

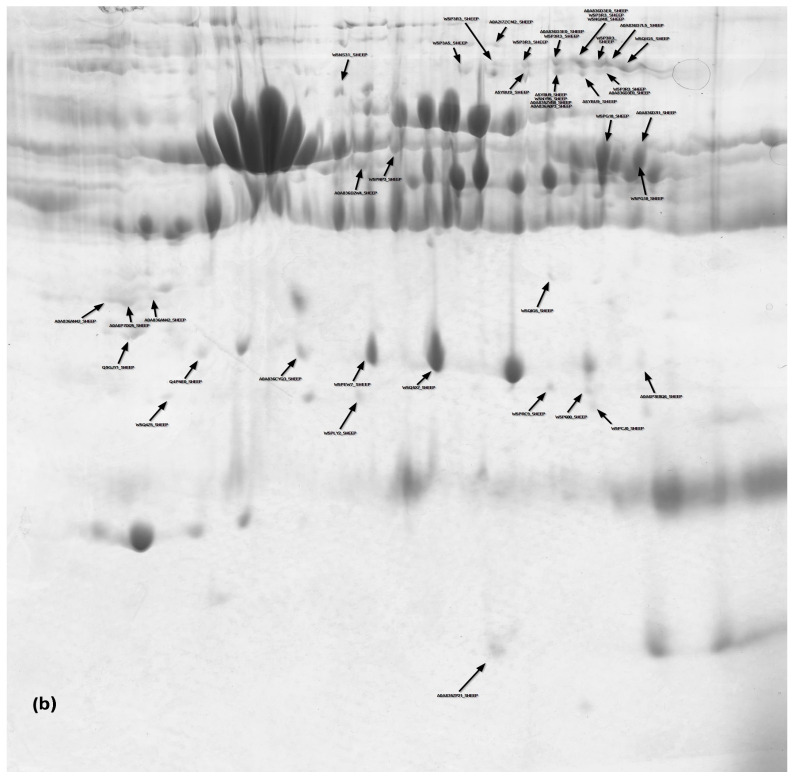

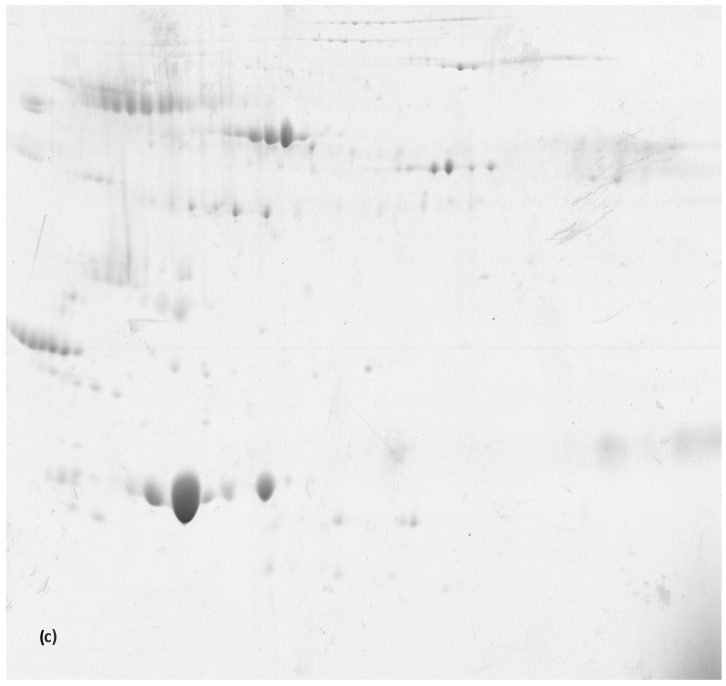

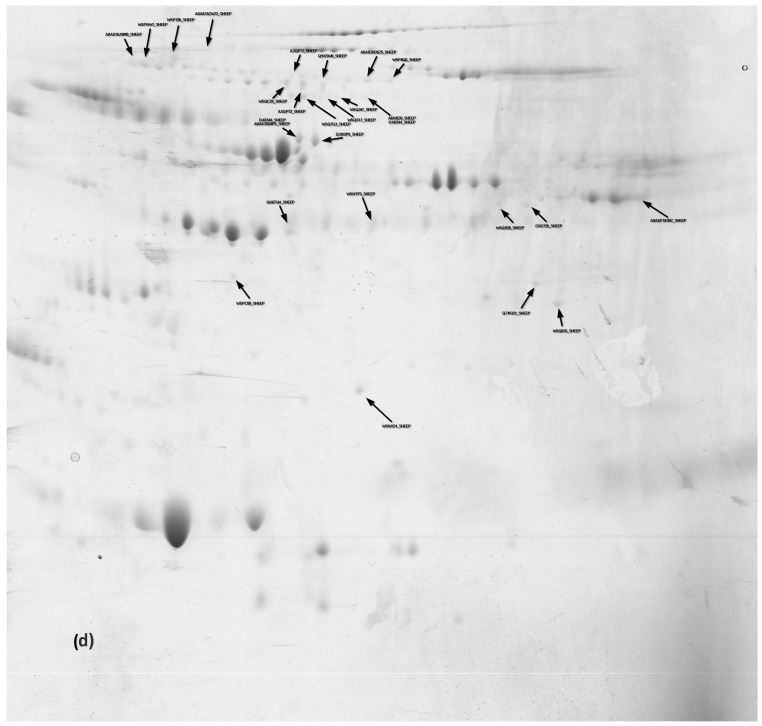
Plasma samples from control ewes (**a**) and melatonin-treated ewes (**b**) and control (**c**) and melatonin ewes (**d**) after applying the ProteoMiner^TM^ enrichment kit at parturition were analyzed via two-dimensional gel electrophoresis (2-DE). The gels were stained with Coomassie blue, and protein spots were excised and further analyzed via matrix-assisted laser desorption ionization time-of-flight mass spectrometry, as described in Section 2. In the gel images, each spot represents a protein. Comparison of protein profiles revealed proteins differentially expressed between the two groups analyzed (arrows). Protein accession numbers used are presented in Table 6 and Table 7.

The rest of the samples were compared to the whole mapping ones as follows: M1-C1 vs. M2, C2, M3, C3, M4 or C4; M5 vs. C5 before and after applying the ProteoMiner^TM^ enrichment kit as well as C3 vs. M3, and the differences were analyzed. Samples M2, C2, M3, C3, M4 and C4 were compared to M1–C1 since it was the control sample of the study prior to melatonin treatment and pregnancy establishment. The findings are presented in Table 2, Table 3, Table 4, Table 5, Table 6 and Table 7, accompanied with the biological process of every unique protein found. Samples C2 and C3 showed no difference in gel spots, with C4 having only two extra proteins compared to these earlier samples, and these were diacylglycerol kinase and GAS2-like protein. In both M4 and C4 samples placental prolactin-related protein 3-like appeared. In total, 53 new proteins appeared at parturition in the M5 sample compared to C5, with seven proteins in M3 compared to C3 after applying the ProteoMiner^TM^ enrichment kit, and 42 proteins derived from the comparison of M1–C1 to M2, M3 or M4. Figure 3 analytically shows the different proteins derived during parturition.

## 4. Discussion

Melatonin exerts antioxidant and immunostimulant action, and it is usually administered to accelerate the onset of the breeding season as a management practice in sheep reproduction. The current proteomics analysis confirms at the molecular level the findings of our previous studies, identifying the positive impact of melatonin administration on pregnant ewes and their newborns [7,8]. These preliminary results elucidate the influence of melatonin during pregnancy in heat stress conditions and at parturition by means of proteomics, revealing the presence of additional proteins. To the best of our knowledge, this is the first time that proteome mapping and screening has been attempted under these conditions in ewes.

Extreme ambient temperature is a well-known promoter of heat stress, causing elevated levels of ROS in animals and cells, disrupting the oxidative balance [33,34]. Elevated ROS cause DNA and protein damage and lipid peroxidation, with primary effects on cell membrane structures and function, and influence endoplasmic reticulum (ER) function by disturbing calcium homeostasis or protein processing/transport [35]. The sample M1-C1 was used as a control to elucidate the proteins that were activated due to the thermal stressor. In order for the cell to alleviate the oxidative stress and survive, it activates the heat shock response (HSR) consisting of heat shock factors and proteins (HSF and HSP, respectively) [34]. The presence of HSF1 and 10 kDa heat shock protein at D0 and D40 in both groups signified the existence of heat stress at the beginning of the experimental period and during the early stages of pregnancy in ewes (see Supplementary Material). HSF1, after its triggering, binds to conserved heat shock-responsive DNA elements (HSEs) promoting heat shock protein-coding genes. Apart from being involved in the heat shock response, HSF1 acts as a regulating factor in various physiological and stress-induced cellular processes and molecular mechanisms. These include protein modification and degradation, cell cycle regulation, programmed cell death, aging, endoplasmic reticulum stress that promotes unfolded protein response (UPR), such as calreticulin and cAMP-responsive element binding protein-like 2, and multidrug resistance as well as immune response, such as defensin 1 and 2, interleukin 3, 5, 8 and 18, coronin-1A and lysozyme. HSP10 is responsible for immune surveillance and the activation of immune response pathways [36]. In the present study, many of the proteins derived from the sample M1–C1 are involved in these pathways, so their activation may be attributed to HSF1 triggering.

In the same sample (M1–C1), additional proteins of interest were found, including clusterin and caspase 3, which seem to collaborate and influence cell death activation and regulate apoptotic mechanisms. Even under normal conditions, they appear to have opposed roles, where caspase 3 regulates apoptosis, whereas clusterin inhibits it [37]. The suppressing effects of heat stress were highlighted by the presence of cathepsin-D, a protease participating in the degradation of intracellular proteins [38]. Moreover, various stress sensors were found, including NmrA-like family domain-containing protein 1 [39], chloride intracellular channel protein 1 [40] and Parkinson disease protein 7 homolog [41]. To counter oxidative stress, the cell mobilizes proteins with antioxidant properties or proteins that upregulate their production. In the latter category are glutathione peroxidase 1 (GPx) and thioredoxin, which are involved in glutathione (GSH) metabolism [42]. GSH effectively scavenges free radicals and other ROS and RNS (e.g., hydroxyl radical, lipid peroxyl radical, superoxide anion and hydrogen peroxide) directly or indirectly through enzymatic reactions [42]. The action of GSH seems to be indirectly supported by the presence of cystatin A, a thiol proteinase inhibitor [43].

Another noteworthy finding in this screening (sample M1–C1) was the identification of proteins that may affect reproduction. Dehydrogenase/reductase SDR family member 11 is reported to reduce 20_α_-hydroxyprogesterone and progesterone secretions [44], whereas follistatin inhibits the biosynthesis and secretion of pituitary follicle-stimulating hormone (FSH) [45]. These proteins may mediate the negative effects of heat stress at the ovarian level and/or the pituitary–gonadal feedback mechanisms.

However, the cell has finite coping mechanisms, and under a very stressful and persistent stimulant this might lead to cell death. Melatonin exerts protective action on the cell against various stressors, but the proteins that remain under its influence are not well known. During pregnancy, as found in samples M2, M3 and/or M4, 42 new proteins appeared compared to the M1–C1 control one. Some of them appeared in every sample, but most of them appeared only once, hinting that they could be related to the pregnancy stage. At time point D16 (sample M2), ewes were under extreme severe heat stress and melatonin concentration had not reached its peak [46], but the presence of 10 new proteins indicates that melatonin’s influence began even at low concentrations. Of this protein bunch, of significant interest is histone deacetylase (HDAC 1), which was present in the samples from D16 until parturition (M5). This protein might contribute to stress alleviation since it is involved in cell differentiation both in the embryo and maternal organism and in signal transduction specifically related to immune response and histone H3 and H4 deacetylation [47,48]. Studies have proven that a reduction in HDAC 1 concentration is directly linked to abnormalities caused by stress in early life [48]. Moreover, the presence of small integral membrane protein 10-like 1 may play a key role in energy metabolism, since its function is linked to the regulation of adipogenesis and adipose cell proliferation [49]. Energy metabolism is crucial for successful pregnancy and healthy newborns, and its regulation is directed by melatonin [50].

The comparison between M3 and M1–C1 revealed multiple proteins that are involved in endoplasmic reticulum organization and interfere with actin filament, ensuring proper protein folding and functionality. One notable difference between samples M3 and C3, when the ProteoMiner^TM^ enrichment kit was applied, was the absence of hypoxia-inducible factor 1 alpha subunit (HIF-1α) in the M3 sample. Chuffa et al. [36] reported that in ovarian carcinoma, a reduction in HIF-1 signaling and in energy and cellular metabolism pathways emerged after long-term melatonin therapy. The same pattern presented in M3 was found in the M4 sample compared to M1–C1, but in this case, the results were enriched with proteins involved in antioxidant defense. Melatonin, through MT1 and MT2 receptors, upregulates GPx gene expression, leading to elevated GSH concentration in cells [51], in order to form GSH conjugates and reduce hydroperoxides [52]. The detoxification of oxidized glutathione (GSSG) is catalyzed by glutathione transferase (GST), which was present in sample M5. The role of GST is not limited to detoxification but includes peroxidase and isomerase action, protecting cells against H_2_O_2_-induced cell death by inhibiting Jun N-terminal kinase, and binds non-catalytically to a wide range of endogenous and exogenous ligands [52]. Moreover, in the same sample, proteins involved in lipid metabolism and transport had a strong presence. It is well established that melatonin interferes with lipid metabolism, regulating energy metabolism during pregnancy [50] and decreasing lipid peroxidation levels [7]. Overall, melatonin seems to be a regulator of many proteins’ expression, having a key role in embryo development, actin filament along with UPR, immune response and antioxidant status, enhancing the ability of the cell to alleviate oxidative stress caused by heat stress and protein degradation.

Numerous physiological changes occur during gestation in the maternal organism, including the generation of ROS, as a result of proliferation and tissue development. Increased metabolism, high consumption of oxygen and the utilization of fatty acids promote the production of ROS in high concentrations during the second trimester of pregnancy. Accordingly, a similar rise in oxidative stress is observed during the final term of gestation, due to increased insulin resistance, fat catabolism and the release of free fatty acids [1]. Oxidative stress is also present at parturition, as studies have reported that high levels of lipid peroxidation due to elevated levels of malondialdehyde [53,54] affect both ER and UPR, as well as the immune response [55]. The presence of long-chain-fatty-acid-CoA ligase at parturition is in line with the latter statement, as the protein is involved in both the synthesis of cellular lipids and degradation, via beta-oxidation, and may activate diverse saturated, monosaturated and polyunsaturated fatty acids [56]. Moreover, ethylmalonyl-CoA decarboxylase was found in the same sample which decarboxylates ethylmalonyl-CoA, a potentially toxic metabolite, to form butyryl-CoA, implying its involvement in metabolite proofreading [57]. For that reason, cells and the maternal organism have to mobilize molecular mechanisms and proteins with protective roles for successful survival. First and foremost, stress detectors are activated, such as ribonuclease inhibitor, ceruloplasmin and urocortin 3, each of them having a distinct role. Ribonuclease inhibitor inhibits RNASE1 and RNASE2, playing a role in redox homeostasis by regulating GSH expression [58]. Ceruloplasmin in blood serum is linked to iron mobilization because it oxidizes Fe^2+^ to Fe^3+^, without releasing radical oxygen species, thus keeping cell redox homeostasis intact [59]. Finally, urocortin 3 has been reported to improve cellular response to stress and specifically alleviates ER stress [60].

Several proteins playing a role in ER function and structure were discovered at parturition in both groups. For example, protein transport protein SEC23 is a component of the coat protein complex II (COPII), promoting the formation of transport vesicles from the ER to the Golgi complex [61], and is required for the translocation of insulin-induced glucose transporter SLC2A4/GLUT4 to cell membranes [62]. Additionally, the small VCP/p97-interacting protein acts as a negative regulator of the ER-associated ubiquitin-dependent protein catabolic process, inhibiting ER-associated degradation [63] and retrograde protein translocation from the ER to cytosol [64]. Finally, junctophilin 4 belongs to the Junctophilin family, which forms a bridge between the plasma membrane and endoplasmic or sarcoplasmic reticulum in excitable cells, providing a structural foundation for functional cross-talk between the cell surface and intracellular calcium release channels [65]. On top of that, a variety of proteins participating in immune response and cell autophagy were revealed. Secretogranin-2 is involved in the inflammatory response, acting as a positive chemiotactic agent for eosinophils [66] and a negative regulator of the endothelial cell apoptotic process [67]. Another negative regulator of apoptosis is the PCI domain-containing protein 2, as the protein is essential for B-cell survival through the regulation of expression of the cell-cycle checkpoint MAD2L1 protein during B-cell differentiation [68]. In the same category as the previous proteins belongs NCK-associated protein 1-like. However, the protein is responsible for chemiotactic events concerning neutrophils, B and T cells, lymphocyte, and erythrocyte differentiation and mTORC2 signaling [69]. In the mTOR pathway, folliculin is involved in causing its downregulation as a response to stress [70], promoting autophagy [71] and participating in the regulation of glycolysis by binding to lactate dehydrogenase LDHA, acting as an uncompetitive inhibitor [72]. The presence of these proteins supports mechanisms allowing the cell to retain its viability and the organism to adapt to new conditions.

During highly stressful events such as parturition, cells are not able to survive. For this reason, melatonin has been proposed as an adjunct agent that can enhance cell survival and accelerate apoptotic phenomena in cell populations that are no longer viable. Melatonin is known to regulate redox status and promote inflammatory and immune response to various stimulants, through affinity receptors MT1 and MT2, which belong to the G-coupled protein receptor family [38]. The results of the current study revealed the existence of 53 additional proteins in the melatonin group, which can be divided into three major categories: immune and inflammatory response, cell cycle regulation and processes supporting protein and ion transportation.

Cesário and colleagues [38] attempted to elucidate how melatonin influences ovarian cancer and found that melatonin upregulates proteins participating in immune response, cellular response to stress and respiratory electron transport, which is in accordance with the current results. Specifically, succinate dehydrogenase complex subunit A flavoprotein is involved in mitochondrial electron transport, playing a key role in the respiratory chain and energy metabolism in cells [73]. In the same study, it was stated that melatonin increases the presence of mitochondrial ATP synthase, which is responsible for ATP production through the driving force of proton generation [38,74]. The latter is supported by the presence of ATP synthase protein 8 in the melatonin group at parturition (sample M5). Finally, in the same group, galectin 1 was downregulated by the presence of melatonin, resulting in the inhibition of the MAPK and JNK/P38 signaling pathway [38]. Galectin was present in the control sample in the current findings, but we were unable to confirm its downregulation by melatonin treatment due to the limitations of the study. Another research group reported that melatonin suppresses the expression of NF-κB antiapoptotic target genes [75]. This finding is in line with the current results because of the presence of histone deacetylase, which is a negative regulator of I-kappaB kinase/NF-kappaB signaling and was present only in the melatonin samples [47,48].

The exogenous administration of melatonin implants in ewes is known to keep melatonin’s concentration stable and higher than its peak at night in normal conditions [46]. Thus, it can be safely assumed that proteins normally upregulated at night are upregulated all day because of the implants. Studies in rats showed that vimentin is one of the proteins that reaches its peak at night because of melatonin and has a multifunctional role [76]. Indeed, vimentin, which was expressed only in the melatonin group, is located in the nucleus, mitochondria and ER and shows antioxidant properties and involvement in actin filament and signal transduction [77]. Vimentin also collaborates with aurora kinase B for vimentin’s phosphorylation, so it controls vimentin filament segregation in the cytokinetic process, whereas histone H3 is phosphorylated at “Ser-10” and “Ser-28” during mitosis [78].

Apart from its protective role in the cell, melatonin plays a key role in parturition. Studies conducted in humans found that melatonin upregulates connexin 43, a gap junction protein that promotes myometrial contractions, along with oxytocin [79], which is in accordance with the current findings. Finally, an odd result was the presence of HSP70 at parturition in the melatonin group, with heat stress being absent. As stated earlier, the heat response occurs not only under the thermal stressor, but following an extremely stressing stimulus, such as parturition [36]. HSP70 belongs to heat shock proteins, a group of molecular chaperones that prevent the aggregation of non-specific proteins and help cellular proteins attain their native structure to maintain cellular homeostasis [80], highlighting again the protective role of melatonin. The current experimental design and promising proteomics results supported a need for further evaluation. A new study using LC/MS-MS techniques and the same protocol has already been conducted, and its results are still to be evaluated for article preparation.

## 5. Conclusions

The findings from this study highlighted the detrimental effects of heat stress during the first critical stages of pregnancy and how they can be reversed using exogenous melatonin administration. Notably, proteomics meticulously identified several proteins whose expressions are upregulated by melatonin and aim to protect cells and the maternal organism during gestation and parturition, emphasizing the significance of melatonin as an adjunct regimen.

## Figures and Tables

**Table 1:** Mean rectal temperature (°C) and breathing rate (breaths·min^−1^) of ewes throughout the first 90 days of the study.

Group	Time	Days of Experimental Period								
		D0–D10	D11–D20	D21–D30	D31–D40	D41–D50	D51–D60	D61–D70	D71–D80	D81–D90
Rectal temperature (°C)										
M	10:00	39.01 ± 0.04	39.12 ± 0.04	39.25 ± 0.03	39.19 ± 0.03	39.10 ± 0.04	39.15 ± 0.04	39.09 ± 0.04	39.07 ± 0.04	38.12 ± 0.06
	16:00	39.62 ± 0.08	39.85 ± 0.03	39.81 ± 0.06	39.96 ± 0.06	40.19 ± 0.05	39.91 ± 0.08	40.07 ± 0.07	39.45 ± 0.02	38.53 ± 0.07
C	10:00	39.17 ± 0.04	39.14 ± 0.06	39.10 ± 0.05	39.17 ± 0.03	39.25 ± 0.05	39.27 ± 0.04	39.26 ± 0.04	39.23 ± 0.04	38.33 ± 0.05
	16:00	39.9 ± 0.02	40.06 ± 0.01	40.03 ± 0.01	40.14 ± 0.01	40.35 ± 0.02	39.79 ± 0.02	39.15 ± 0.01	39.78 ± 0.05	38.62 ± 0.09
Breathing rate (breaths min^−1^)										
M	10:00	53.75 ± 2.7	54.4 ± 2.2	54.7 ± 2.1	56.7 ± 1.6	54.85 ± 1.6	59.8 ± 1.5	61.5 ± 2.2	55.3 ± 1.2	40.6 ± 2.3
	16:00	88.05 ± 2.8	81.75 ± 2.8	91.1 ± 2.4	86.35 ± 1.7	88 ± 1.8	94.4 ± 1.3	94.5 ± 1.8	87.4 ± 2.9	45.2 ± 1.9
C	10:00	64.6 ± 0.8	64.9 ± 1.1	64.7 ± 1.2	65.4 ± 0.7	67.7 ± 0.8	66.1 ± 0.6	67.1 ± 0.8	56.8 ± 0.8	41.4 ± 2.1
	16:00	98.6 ± 1.1	103.8 ± 1.6	102.6 ± 2.2	101.6 ± 0.8	117.1 ± 4.1	111.5 ± 1.8	108.3 ± 2.1	93 ± 2.7	46 ± 0.8

**Table 2:** The different proteins derived from a comparison between M2 and M1–C1. The table provides their identities, biological process, protein coverage, molecular weight, theoretical pI and Mascot score as calculated with PDQuest 8.0 software.

Full Protein Name	Accession	Biological Process	Mascot Score	MS Coverage	Protein MW	pI Value
Anaphase-promoting complex subunit 10	A0A835ZLF7_SHEEP	anaphase-promoting complex-dependent catabolic process; cell cycle; cell division	42	18	17,605.00	10.10
Arylamine N-acetyltransferase	A0A6P7DI25_SHEEP	xenobiotic metabolic process	41	8	34,403.00	5.20
Centromere protein P	W5PEF0_SHEEP	CENP-A containing chromatin assembly	44	17	33,114.00	4.90
Glucosylceramidase	A0A291RBV4_SHEEP	cholesterol metabolic process; sphingolipid metabolic process	42	12	8476.00	6.50
Histone deacetylase	W5QIG5_SHEEP	circadian regulation of gene expression; embryonic digit morphogenesis; endoderm development; epidermal cell differentiation; negative regulation of I-kappaB kinase/NF-kappaB signaling; neuron differentiation; oligodendrocyte differentiation; positive regulation of oligodendrocyte differentiation; positive regulation of signaling receptor activity; regulation of endopeptidase activity; negative regulation by host of viral transcription; histone H3 and H4 deacetylation	43	8	55,641.00	5.20
IQ motif and Sec7 domain ArfGEF 2	W5PG04_SHEEP	regulation of ARF protein signal transduction	42	4	143,693.00	9.30
Phosphoinositide 5-phosphatase	W5PZG5_SHEEP	phosphatidylinositol dephosphorylation; signal transduction	41	10	105,788.00	6.30
RNA helicase	A0A836A0L4_SHEEP	antiviral innate immune response; defense response to bacterium and virus; mRNA processing; mRNA splicing, via spliceosome; positive regulation of I-kappaB kinase/NF-kappaB signaling; response to alkaloid; response to toxic substance; RNA splicing	41	5	142,083.00	7.00
Small integral membrane protein 10-like 1	W5QGF7_SHEEP	regulation of adipogenesis; adipose cell proliferation	42	19	7392.00	10.70
Small ubiquitin-related modifier	W5PRT2_SHEEP	protein sumoylation; negative regulation of DNA binding; regulation of protein localization to nucleus	56	32	11,808.00	5.20

**Table 3:** The different proteins derived from a comparison between M3 and M1-C1. The table provides their identities, biological process, protein coverage, molecular weight, theoretical pI and Mascot score as calculated with PDQuest 8.0 software.

Full Protein Name	Accession	Biological Process	Mascot Score	MS Coverage	Protein MW	pI Value
BARX homeobox 2	W5PVP0_SHEEP	cartilage condensation; DNA-templated transcription; DNA-templated transcription elongation; myotube differentiation; regulation of transcription by RNA polymerase II; skeletal muscle cell differentiation; skeletal muscle cell differentiation	42	15	32,552.00	10.10
Beta-2-glycoprotein 1	W5Q268_SHEEP	blood coagulation, intrinsic pathway; negative regulation of angiogenesis; negative regulation of endothelial cell migration and proliferation; negative regulation of fibrinolysis; negative regulation of myeloid cell apoptotic process; negative regulation of smooth muscle cell apoptotic process; plasminogen activation; positive regulation of lipoprotein lipase activity; triglyceride metabolic process	85	37	39,442	9.66
C2 domain-containing protein	A0A836A9U4_SHEEP	exocytosis	42	18	29,554	9.43
Centromere protein X	W5Q881_SHEEP	kinetochore assembly; DNA repair	43	34	9557.00	5.50
FK506-binding protein-like	A0A836CTK1_SHEEP	endocytosis	40	6	38,194.00	5.70
Glutaredoxin domain-containing protein	A0A836CUB0_SHEEP	nucleobase-containing small molecule interconversion; positive regulation of membrane potential; positive regulation of sodium ion transmembrane transporter activity; protein deglutathionylation	42	20	8226	6.49
HID1 domain-containing	W5PPI7_SHEEP	vesicle trafficking within the trans-Golgi network	42	8	86,737.00	5.70
Histone deacetylase	W5QIG5_SHEEP	circadian regulation of gene expression; embryonic digit morphogenesis; endoderm development; epidermal cell differentiation; negative regulation of I-kappaB kinase/NF-kappaB signaling; neuron differentiation; oligodendrocyte differentiation; positive regulation of oligodendrocyte differentiation; positive regulation of signaling receptor activity; regulation of endopeptidase activity; negative regulation by host of viral transcription; histone H3 and H4 deacetylation	41	9	55,641.00	5.20
Kelch-like protein 1	W5Q6M6_SHEEP	actin cytoskeleton organization; cerebellar Purkinje cell layer development; dendrite development	41	8	17,817.00	9.60
Lipocalin/cytosolic fatty-acid binding domain-containing protein	W5PZN0_SHEEP	small hydrophobic molecule transportation	42	19	20,224.00	5.20
Neurogenic differentiation factor	W5PF29_SHEEP	cell differentiation; dentate gyrus development	41	10	39,105.00	9.50
PRDM9	A0A1B1SDL6_SHEEP	double-strand break repair involved in meiotic recombination; male/female gamete generation; histone methylation; homologous; chromosome pairing at meiosis; meiotic gene conversion; negative regulation of apoptotic process; positive regulation of fertilization; positive regulation of reciprocal meiotic recombination; regulation of DNA-templated transcription	44	20	27,097.00	11.40
RING-type domain-containing protein	W5P2I5_SHEEP	embryonic brain development; endoplasmic reticulum organization; neuron differentiation; protein autoubiquitination; protein homooligomerization; regulation of cell cycle; response to hydroperoxide	41	37	7992.00	7.70
Shroom family member 2	W5PK86_SHEEP	actin filament organization	42	5	152,796	8.75
Tubulin/FtsZ two-layer sandwich domain-containing protein	A0A836CW31_SHEEP	microtubule-based process	42	18	44,502.00	5.50
valine–tRNA ligase	W5P3E7_SHEEP	valyl-tRNA aminoacylation	43	8	75,540.00	9.80

**Table 4:** The different proteins derived from a comparison between M4 and M1-C1. The table provides their identities, biological process, protein coverage, molecular weight, theoretical pI and Mascot score as calculated with PDQuest 8.0 software.

Full Protein Name	Accession	Biological Process	Mascot Score	MS Coverage	Protein MW	pI Value
Anaphylatoxin-like domain-containing protein	W5NRH2_SHEEP	complement activation, classical pathway; inflammatory response; innate immune response	69	37	15,047	7.69
Apolipoprotein A4	W5NWM2_SHEEP	lipid transport; lipoprotein metabolic process	85	32	41,512	5.46
Apolipoprotein E	W5PI61_SHEEP	lipid transport; lipoprotein metabolic process	44	11	36,216.00	5.60
BARX homeobox 2	W5PVP0_SHEEP	cartilage condensation; DNA-templated transcription; DNA-templated transcription elongation; myotube differentiation; regulation of transcription by RNA polymerase II; skeletal muscle cell differentiation; skeletal muscle cell differentiation	42	9	32,552.00	10.10
Core shell protein Gag P30 domain-containing protein	W5Q4W1_SHEEP	virion assembly	42	9	53,882	9.31
EF-hand domain-containing protein	A0A836D0W4_SHEEP	cerebral cortex cell migration; cilium-dependent cell motility; mitotic cytokinesis; mitotic spindle organization; regulation of cell division	43	12	26,738.00	5.00
Glutaredoxin domain-containing protein	A0A836CUB0_SHEEP	nucleobase-containing small molecule interconversion; positive regulation of membrane potential; positive regulation of sodium ion transmembrane transporter activity; protein deglutathionylation	40	20	8226	6.49
Glutathione transferase	A0A6P7D2D4_SHEEP	glutathione derivative biosynthetic process; glutathione metabolic process; prostaglandin metabolic process; xenobiotic metabolic process	43	21	26,673	6.28
Histone deacetylase	W5QIG5_SHEEP	circadian regulation of gene expression; embryonic digit morphogenesis; endoderm development; epidermal cell differentiation; negative regulation of I-kappaB kinase/NF-kappaB signaling; neuron differentiation; oligodendrocyte differentiation; positive regulation of oligodendrocyte differentiation; positive regulation of signaling receptor activity; regulation of endopeptidase activity; negative regulation by host of viral transcription; histone H3 and H4 deacetylation	44	8	55,641.00	5.20
Integrin beta	W5PS30_SHEEP	cell adhesion; integrin-mediated signaling pathway; phagocytosis	41	9	82,901	6.26
Multivesicular body subunit 12A	W5Q5E2_SHEEP	protein transport	42	22	29,419.00	10.40
ORM1-like protein	A0A6P3E1H8_SHEEP	cellular lipid metabolic process; regulation of ceramide biosynthetic process	43	21	17,350.00	9.90
Protein Shroom 2	A0A835ZLJ3_SHEEP	actin filament organization	44	8	163,412	6.26
Ribonuclease inhibitor	W5PBY5_SHEEP	cell migration; mRNA catabolic process; regulation of actin cytoskeleton reorganization; regulation of angiogenesis; regulation of Arp2/3 complex-mediated actin nucleation	42	21	11,496.00	4.30
Trefoil factor 3	Q30DP5_SHEEP	maintenance of gastrointestinal epithelium; regulation of glucose metabolic process; response to peptide hormone; wound healing	41	15	4940.00	4.20
Tyrosine aminotransferase	A0A6P7ERC0_SHEEP	biosynthetic process; L-phenylalanine catabolic process; tyrosine catabolic process	43	6	50,700.00	5.80

**Table 5:** The different proteins derived from a comparison between M3 and C3 after applying the ProteoMiner^TM^ enrichment kit. The table provides their identities, biological process, protein coverage, molecular weight, theoretical pI and Mascot score as calculated with PDQuest 8.0 software.

Full Protein Name	Accession	Biological Process	Mascot Score	MS Coverage	Protein MW	pI Value
BPTI/Kunitz inhibitor domain-containing protein	A0A836CZM4_SHEEP	negative regulation of peptidase activity	41	22	13,735.00	10.40
EF-hand domain-containing protein	A0A836D0W4_SHEEP	cerebral cortex cell migration; cilium-dependent cell motility; mitotic cytokinesis; mitotic spindle organization; regulation of cell division	42	24	8862.00	5.10
Glycerol-3-phosphate dehydrogenase [NAD(+)]	W5Q983_SHEEP	carbohydrate metabolic process; glycerol-3-phosphate catabolic process	41	5	38,093.00	6.40
Integrin beta	W5PS30_SHEEP	cell adhesion; integrin-mediated signaling pathway; phagocytosis	45	8	82,901.00	6.30
Interleukin-1	W5QI50_SHEEP	immune response; inflammatory response	41	14	17,672.00	9.20
Methyl-CpG-binding domain protein 3-like 3	W5Q4M5_SHEEP	aging; brain development; embryonic organ development; DNA methylation-dependent heterochromatin formation; heart development; histone acetylation and deacetylation; in utero embryonic development; negative regulation of DNA-templated transcription; negative regulation of transcription by RNA polymerase II; positive regulation of DNA-templated transcription; regulation of cell fate specification; regulation of DNA methylation; regulation of stem cell differentiation; response to estradiol; response to nutrient levels; tissue development	45	10	21,834.00	11.70
V-kit Hardy–Zuckerman 4 feline sarcoma viral oncoprotein	A0A0C5G4P5_SHEEP	protein phosphorylation	43	25	12,170.00	5.20

**Table 6:** The different proteins derived from a comparison between M5 and C5. The table provides their identities, biological process, protein coverage, molecular weight, theoretical pI and Mascot score as calculated with PDQuest 8.0 software.

Full Protein Name	Accession	Biological Process	Mascot Score	MS Coverage	Protein MW	pI Value
Arylamine N-acetyltransferase	A0A6P7DI25_SHEEP	xenobiotic metabolic process	43	8	34,403.00	5.20
ATP synthase protein 8	A0A2I7ZCM2_SHEEP	proton motive force-driven ATP synthesis	41	12	7875.00	10.00
Baculoviral IAP repeat containing 5	W5P600_SHEEP	cell division; establishment of chromosome localization; G2/M transition of mitotic cell cycle; meiosis I; microtubule cytoskeleton organization; mitotic cell cycle; mitotic cytokinesis; mitotic spindle assembly check-point signaling; mitotic spindle midzone assembly; mitotic spindle organization; negative regulation of apoptotic process; negative regulation of cysteine-type endopeptidase activity involved in apoptotic process; negative regulation of DNA-templated transcription; negative regulation of neuron apoptotic process; negative regulation of neuron apoptotic process; positive regulation of cell cycle; positive regulation of exit from mitosis; positive regulation of mitotic cell cycle; positive regulation of mitotic cell cycle spindle assembly checkpoint; positive regulation of mitotic cytokinesis; positive regulation of mitotic sister chromatid separation; positive regulation of protein phosphorylation; positive regulation of protein ubiquitination; protein-containing complex localization; regulation of insulin secretion involved in cellular response to glucose stimulus; regulation of type B pancreatic cell proliferation	40	21	16,660.00	5.40
C1q domain-containing protein	A0A836D7I1_SHEEP	serum complement system activation	43	12	26,485.00	10.20
C3/C5 convertase	A5YBU9_SHEEP	complement activation; proteolysis; innate immune response	83	11	86,744.00	9.10
Centromere protein F	W5PEW7_SHEEP	chromosome segregation; kidney development; metaphase plate congression; negative regulation of DNA-templated transcription; protein transport; regulation of G2/M transition of mitotic cell cycle; regulation of striated muscle tissue development; ventricular system development	45	4	355,326	4.87
Coiled-coil domain-containing 187	W5P3A5_SHEEP	microtubule anchoring	42	4	112,761.00	11.70
Collagen alpha-1 (XX) chain	W5PNP3_SHEEP	collagen trimer	44	14	45,337	7.97
Collectin-10	A0A836A0P3_SHEEP	cell surface pattern recognition receptor signaling pathway; complement activation, lectin pathway; cranial skeletal system development; positive regulation of opsonization; proteolysis	45	15	17,552	10.25
Complement C2	W5NY95_SHEEP	complement activation, classical pathway; innate immune response; proteolysis	64	7	140,645	9.19
Connexin 43	Q9GJY1_SHEEP	cell communication	42	24	14,101.00	10.00
Cortactin	W5NQM8_SHEEP	brain development; regulation of modification of postsynaptic actin cytoskeleton; regulation of synapse organization	43	11	53,261	5.16
Discoidin, CUB and LCCL domain-containing 1	W5PG18_SHEEP	intracellular receptor signaling pathway; negative regulation of cell growth; wound healing	41	11	73,223.00	9.80
Epithelial cell transforming 2-like	W5NS31_SHEEP	positive regulation of GTPase activity; regulation of cytokinesis; actomyosin contractile ring assembly	40	11	105,770	9.11
Eukaryotic translation initiation factor 3 subunit H	W5PLY2_SHEEP	formation of cytoplasmic translation initiation complex	44	10	32,619.00	8.80
F-box domain-containing protein	A0A836AN42_SHEEP	protein ubiquitination	42	24	31,339	10.57
Fibrinogen silencer binding protein	W5PCJ0_SHEEP	identical protein binding activity	45	13	34,811.00	6.83
IKAROS family zinc finger 3	W5PRC9_SHEEP	B cell differentiation; positive regulation of transcription by RNA polymerase II; regulation of apoptotic process; regulation of B cell proliferation; regulation of lymphocyte differentiation; regulation of transcription by RNA polymerase II; response to bacterium; T cell differentiation; mesoderm development	43	6	59,046.00	6.10
Keratin, type II cytoskeletal 3-like	W5Q5X7_SHEEP	epithelial cell differentiation; intermediate filament cytoskeleton organization	42	4	50,478.00	5.60
MARVEL domain-containing protein	A0A836D7L5_SHEEP	cell cycle; myelination				
MHC class I-like antigen recognition-like domain-containing protein	W5NX45_SHEEP	antigen processing and presentation of endogenous peptide antigen via MHC class I via ER pathway, TAP-independent; antigen processing and presentation of endogenous peptide antigen via MHC class Ib; immune response; positive regulation of T cell-mediated cytotoxicity	41	7	32,714.00	4.90
Plasminogen	A0A836D3E0_SHEEP	blood coagulation; proteolysis	177	25	93,859.00	7.80
Protein deglycase	A0A6P3E8Q6_SHEEP	autophagy; single fertilization	40	12	20,222.00	7.70
Sarcosine dehydrogenase, mitochondrial	A0A836D2W4_SHEEP	choline catabolic process; glycine biosynthetic process; sarcosine catabolic process; tetrahydrofolate interconversion	42	4	100,464.00	6.60
SLC9A3 regulator 2	W5Q4Z5_SHEEP	proteolysis	42	18	18,981.00	5.85
Synaptonemal complex central element protein 1	A0A835ZP21_SHEEP	synaptonemal complex assembly	42	18	24,827.00	5.50
Voltage-gated potassium channel Kv2.1	Q4PNE0_SHEEP	action potential; cellular response to glucose stimulus; exocytosis; negative regulation of insulin secretion; positive regulation of long-term synaptic depression; positive regulation of protein targeting to membrane; protein homooligomerization; regulation of action potential; regulation of monoatomic ion transmembrane transport	41	14	6715.00	7.50

**Table 7:** The different proteins derived from a comparison between M5 and C5 after applying the ProteoMiner^TM^ enrichment kit. The table provides their identities, biological process, protein coverage, molecular weight, theoretical pI and Mascot score as calculated with PDQuest 8.0 software.

Full Protein Name	Accession	Biological process	Mascot Score	MS Coverage	Protein MW	pI Value
ABC transmembrane type-1 domain-containing protein	W5NTP3_SHEEP	transmembrane transport	41	26	9740.00	5.60
Acyl-CoA dehydrogenase/oxidase C-terminal domain-containing protein	W5Q8A9_SHEEP	fatty acid beta-oxidation	44	23	9369.00	6.10
Aldo-keto reductase family 1 member B	W5PC00_SHEEP	C21-steroid hormone biosynthetic process; negative regulation of cellular apoptotic process; hyperosmotic salinity response; epithelial cell maturation; carbohydrate and daunarubicin and doxorubicin metabolic process	40	9	36,292.00	5.90
Beta defensin OA300	W5P8Q6_SHEEP	defense response to bacterium	42	40	7966.00	11.20
Bystin-like	W5NXZ4_SHEEP	trophectodermal cell differentiation; stem cell proliferation; maturation of SSU-rRNA from tricistronic rRNA transcript (SSU-rRNA, 5.8S rRNA, LSU-rRNA); rRNA processing	48	7	42,623.00	6.40
Complement C1r	W5P336_SHEEP	complement activation; innate immune response; proteolysis	49	7	81,681.00	5.60
Complement component 3d	A6NBZ0_SHEEP	stimulation of antigen presentation; maintenance B cell memory	72	15	34,420	6.21
Complement component C3	O46544_SHEEP	immune response	82	17	39,826	6.12
Ethanolamine-phosphate cytidylyltransferase	A0A836A9M0_SHEEP	phosphatidylethanolamine biosynthetic process	44	1	79,699.00	7.90
Galactose-3-O-sulfotransferase 4	W5Q7G3_SHEEP	glycolipid biosynthetic process	44	4	52,506.00	10.50
Golgi membrane protein 1	A0A836D8P5_SHEEP	protein folding	45	14	47,089	4.68
Heat shock 70 kDa protein 4	A0A836AJ35_SHEEP	chaperone-mediated protein complex assembly; protein insertion into mitochondrial outer membrane	46	22	40,883.00	9.30
Histone deacetylase	W5QIG5_SHEEP	circadian regulation of gene expression; embryonic digit morphogenesis; endoderm development; epidermal cell differentiation; negative regulation of I-kappaB kinase/NF-kappaB signaling; neuron differentiation; oligodendrocyte differentiation; positive regulation of oligodendrocyte differentiation; positive regulation of signaling receptor activity; regulation of endopeptidase activity; negative regulation by host of viral transcription; histone H3 and H4 deacetylation	42	8	55,641.00	5.20
Homer protein	Q9XSW0_SHEEP	G protein-coupled glutamate receptor signaling pathway; negative regulation of calcineurin-NFAT signaling cascade; negative regulation of interleukin-2 production; protein targeting; regulation of postsynaptic neurotransmitter receptor activity; regulation of store-operated calcium entry	45	12	7205.00	10.10
Inter-alpha-trypsin inhibitor heavy chain H4	A0A835ZW72_SHEEP	negative regulation of peptidase activity; hyaluronan metabolic process	48	4	100,701.00	6.00
Metallothionein	W5QAI1_SHEEP	astrocyte activation; cellular response to cadmium and copper and zinc ion and erythropoietin; negative regulation of growth; response to metal ion	46	14	7265.00	10.50
Phorbol-ester/DAG-type domain-containing protein	A0A836D5Z5_SHEEP	protein phosphorylation	47	4	43,929.00	9.20
PR/SET domain 6	W5QC25_SHEEP	histone methylation	42	4	61,824.00	9.50
Pregnancy-associated glycoprotein 5	O02725_SHEEP	proteolysis	47	4	42,729.00	10.00
Procollagen C-endopeptidase enhancer	W5Q517_SHEEP	collagen biosynthetic process; proteolysis	42	4	49,080.00	9.00
Protein kinase C inhibitor KCIP-1 isoform eta	Q7M331_SHEEP	cell proliferation; cell differentiation; cell death	41	57	2531.00	4.40
PX domain-containing protein	A0A6P3E997_SHEEP	protein sorting; vesicular trafficking; phospholipid metabolism	43	13	39,036.00	4.70
Succinate dehydrogenase complex subunit A flavoprotein	A3QP72_SHEEP	mitochondrial electron transport; succinate to ubiquinone; nervous system development; succinate metabolic process	47	19	5144.00	7.60
Trefoil factor 3	Q30DP5_SHEEP	maintenance of gastrointestinal epithelium; regulation of glucose metabolic process; response to peptide hormone; wound healing	42	15	4940.00	4.20
Vimentin	W5PNW7_SHEEP	positive regulation of superoxide anion generation; astrocyte development; Bergmann glial cell differentiation; cellular response to lipopolysaccharide and muramyl dipeptide and type II interferon; in utero embryonic development; intermediate filament organization; negative regulation of neuron projection development; positive regulation of collagen biosynthetic process; positive regulation of translation; regulation of mRNA stability; SMAD protein signal transduction	48	12	53,711.00	4.90
Zinc finger protein	Q66TQ4_SHEEP	transcription regulation	49	11	6230.00	10.50

## Data Availability

Data are available in the Supplementary Materials.

## Supplementary Materials

The following supporting information can be downloaded at: https://www.mdpi.com/article/10.3390/ani14030400/s1.
